# Widespread failure to complete meiosis does not impair fecundity in parthenogenetic whiptail lizards

**DOI:** 10.1242/dev.141283

**Published:** 2016-12-01

**Authors:** Aracely A. Newton, Robert R. Schnittker, Zulin Yu, Sarah S. Munday, Diana P. Baumann, William B. Neaves, Peter Baumann

**Affiliations:** 1 Stowers Institute for Medical Research, Kansas City, MO 64110, USA; 2 Howard Hughes Medical Institute, Kansas City, MO 64110, USA; 3 Department of Molecular and Integrative Physiology, University of Kansas Medical Center, Kansas City, KS 66160, USA

**Keywords:** Parthenogenesis, *Aspidoscelis*, Meiosis, Endoreplication, Synaptonemal complex

## Abstract

Parthenogenetic species of whiptail lizards in the genus *Aspidoscelis* constitute a striking example of speciation by hybridization, in which first-generation hybrids instantly attain reproductive isolation and procreate as clonal all-female lineages. Production of eggs containing a full complement of chromosomes in the absence of fertilization involves genome duplication prior to the meiotic divisions. In these pseudo-tetraploid oocytes, pairing and recombination occur exclusively between identical chromosomes instead of homologs; a deviation from the normal meiotic program that maintains heterozygosity. Whether pseudo-tetraploid cells arise early in germ cell development or just prior to meiosis has remained unclear. We now show that in the obligate parthenogenetic species *A. neomexicana* the vast majority of oocytes enter meiosis as diploid cells. Telomere bouquet formation is normal, but synapsis fails and oocytes accumulate in large numbers at the pairing stage. Pseudo-tetraploid cells are exceedingly rare in early meiotic prophase, but they are the only cells that progress into diplotene. Despite the widespread failure to increase ploidy prior to entering meiosis, the fecundity of parthenogenetic *A. neomexicana* is similar to that of *A. inornata*, one of its bisexual ancestors.

## Abstract (precis)

**Summary:** The fecundity of unisexual whiptail lizards is similar to bisexual relatives despite pseudo-tetraploid oocytes being exceedingly rare and diploid oocytes failing to proceed past the pairing stage of meiosis.

## INTRODUCTION

Interspecific hybridization is a common phenomenon in plants and animals (Mallet, 2007). If the parental species are closely related, first generation hybrids often retain the ability to interbreed with one or both of the parental species. Hybridization between more divergent species commonly results in embryonic lethality or hybrid sterility. In some cases, hybridization causes reproductive isolation and formation of a new species of hybrid origin (Mavárez et al., 2006). Parthenogenetic species of lizards constitute an interesting and unusual case of hybrid speciation (Avise, 2008). The genus *Aspidoscelis*, partitioned from *Cnemidophorus* in 2002 (Reeder et al., 2002), consists of bisexual and parthenogenetic species, with each diploid parthenogenetic species being the result of an ancestral hybridization event between members of two sexually reproducing species (Lowe and Wright, 1966; Neaves, 1969; Neaves and Gerald, 1968; Reeder et al., 2002). In some cases, secondary and tertiary hybridization events then gave rise to triploid and tetraploid species that also reproduce by parthenogenesis (Cole et al., 2014; Lutes et al., 2011; Neaves and Gerald, 1969; Pennock, 1965). Interspecific hybridization endowed the first generation hybrids with a high degree of heterozygosity, which clonal reproduction has maintained over many generations. This long-term maintenance of heterozygosity and associated hybrid vigor is widely regarded as a crucial asset that permits unisexual species to compete favorably with sexually reproducing relatives.

**Fig. 1.**
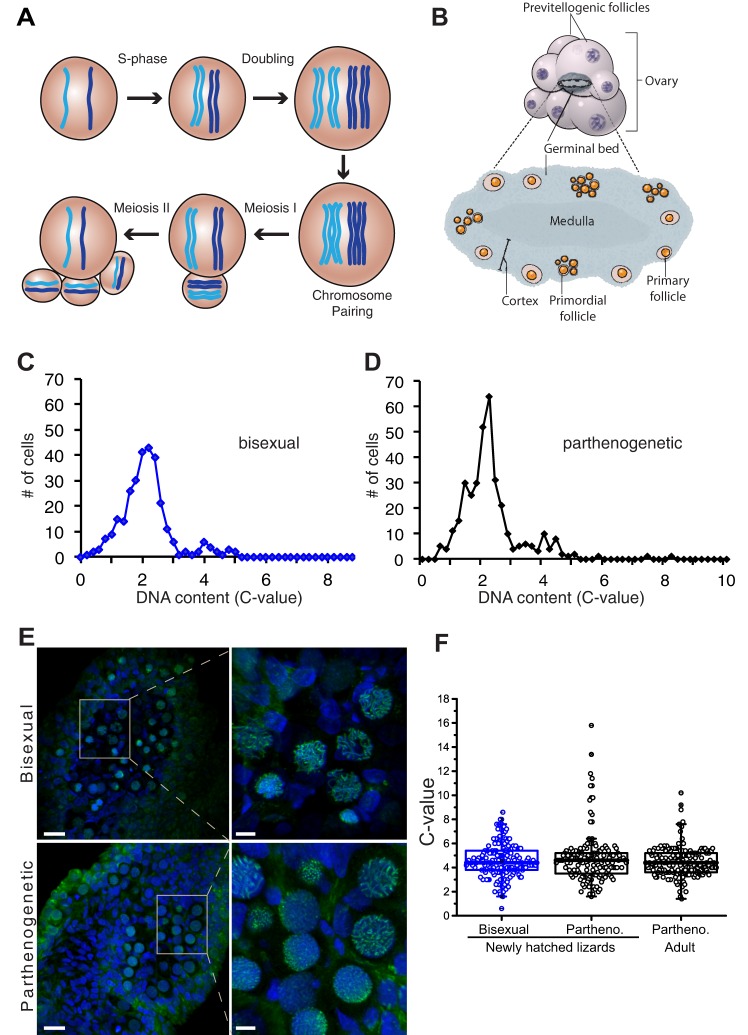
**DNA content in oocytes and somatic cells in the germinal bed.** (A) Schematic of meiosis in parthenogenetic whiptail lizards. A single pair of homologous chromosomes are shown in light and dark blue. Following premeiotic S-phase and an additional doubling, identical chromosomes – not homologs – pair and recombine. The two meiotic divisions give rise to a diploid oocyte and three polar bodies. (B) Schematic of ovaries comprised of previtellogenic follicles and the germinal bed, which contains the earlier stages of germ cell development including primordial and early primary follicles embedded in connective tissue within its cortex. (C) DNA content analysis for germinal bed cells from the bisexual species *A. inornata* (*n*=292). Tissues were DAPI-stained and DNA content measured based on fluorescence intensity of individual nuclei. (D) DNA content analysis as in C for germinal bed from parthenogenetic *A. neomexicana* (*n*=351). (E) Germinal beds of *A. inornata* (top) and *A. neomexicana* (bottom) stained with DAPI (blue) and anti-SYCP3 serum (green). The boxed area in the left images is enlarged on the right. Scale bars: 20 µm, left; 5 µm, right. (F) DNA content for oocytes from germinal beds of newly hatched *A. inornata* (left, *n*=167), newly hatched *A. neomexicana* (middle, *n*=148), and adult *A. neomexicana* ranging in age from 16-22 months (right, *n*=179).

Whereas interspecific hybridization is a widespread phenomenon in fish, amphibians and reptiles, parthenogenetic species have only arisen in a small subset of taxa (Neaves and Baumann, 2011; Vrijenhoek et al., 1989). A significant obstacle in switching from sexual to parthenogenetic reproduction is the requirement to modify oogenesis to generate eggs that contain a full complement of chromosomes in the absence of fertilization. Examination of the DNA content of oocytes from parthenogenetic whiptail lizards provided conclusive evidence for previtellogenic follicles containing twice the number of chromosomes compared with follicles from closely related bisexual species (Cuellar, 1971; Lutes et al., 2010). By increasing the number of chromosomes to pseudo-tetraploidy prior to meiosis, the normal reductional division produces diploid rather than haploid eggs (Fig. 1A). The use of *in situ* hybridization probes that distinguish between homologous chromosomes revealed that pairing and recombination occur exclusively between genetically identical chromosomes instead of homologs (Lutes et al., 2010). This important deviation from the normal meiotic program explains the long-term maintenance of heterozygosity in parthenogenetic species.

Discerning the mechanism that underlies the transient genome duplication prior to the meiotic divisions is crucial for understanding the regulatory processes that trigger parthenogenesis in hybrids. In principle, two rounds of replication without intervening mitosis (endoreplication) would generate a cell of the required DNA content. If such an event occurs early in development, a tetraploid germ line will reside in an otherwise diploid animal. Alternatively, if the additional doubling of DNA content directly precedes meiosis, most of the germ line will be diploid and only cells in prophase I of meiosis will be pseudo-tetraploid. In this study, we have examined DNA content of cells in the germinal beds of *A. neomexicana*, a diploid parthenogenetic species, and *A. inornata*, one of its bisexual progenitor species. Surprisingly, we found that diploid oocytes enter the early stages of prophase I in large numbers, but fail to progress beyond the pairing stage in *A. neomexicana*. Rare pseudo-tetraploid cells progress through meiosis and produce diploid gametes that spontaneously develop in the absence of fertilization.

## RESULTS

To gain insights into the timing and mechanism underlying the ploidy increase in the germ line, we examined cells in the germinal beds (GB) of bisexual *A. inornata* and parthenogenetic *A. neomexicana*. The GB is found within the ovaries of lower vertebrates and contains a variety of different cell types including primordial and primary follicles (Fig. 1B). Primary follicles transition through the early stages of prophase I into early diplotene, when they leave the cortex of the GB and become previtellogenic follicles that increase in size from ∼40 µm to ∼3 mm prior to undergoing diakinesis. Prior reports of a transient doubling in DNA content from 4C to 8C was exclusively based on DNA quantification and the counting of bivalents in nuclei isolated from previtellogenic follicles (Cuellar, 1971; Lutes et al., 2010).

### Measurement of germ cell DNA content

To determine whether the additional doubling in chromosomal DNA occurs early or late in germ cell development, we examined ∼300 randomly chosen cells in whole-mount DAPI-stained germinal beds of bisexual and parthenogenetic animals by image cytometry (Baltus et al., 2006). This method was chosen as the number of cells in the ovary is too small for flow cytometry. As somatic cells make up a large portion of the GB (see below) and are predominantly in G1 or G0, we defined the median intensity peak as 2C (Fig. 1C). A second much smaller peak corresponded to twice the intensity, consistent with diploid cells in G2 or meiotic prophase. Surprisingly, the profile from the parthenogenetic animals was very similar to that of the bisexual species (Fig. 1D). Most importantly, no significant peak was observed at 8C, where cells that had undergone endoreplication would be expected. This observation was inconsistent with the presence of a pseudo-tetraploid germ line.

To specifically examine the DNA content of oocytes in prophase of meiosis I, we identified the synaptonemal complex protein-3 (SYCP3) in *Aspidoscelis* based on similarity with sequences from other vertebrates (Fig. S1) and raised antibodies against the recombinant protein. In mammals, SYCP3 antibodies label oocytes from pre-leptotene to pachytene (Dobson et al., 1994; Pfeifer et al., 2003). Immunostaining of germinal beds from newly hatched *A. inornata* and *A. neomexicana* with anti-SYCP3 revealed a subset of germinal bed cells with defined staining (Fig. 1E), closely resembling the patterns of SYCP3 localization in bovine oocytes (Pfeifer et al., 2003).

The mean DNA content of SYCP3-positive cells from *A. inornata* (bisexual) was close to 4C, consistent with diploid cells in meiotic prophase (Fig. 1F). Surprisingly, the same mean DNA content was observed in *A. neomexicana*, indicating that most oocytes enter meiosis without prior doubling of chromosomes in this parthenogenetic species. Notably, 7% of oocytes from the parthenogenetic species, but <1% for the bisexual species, had an apparent DNA content consistent with polyploidy. We wondered whether endoreplication giving rise to 8C oocytes might not occur until animals reach reproductive maturity. However, measurement of oocyte DNA content from adult parthenogenetic lizards also revealed a predominantly 4C DNA content (Fig. 1F). Thus, the ploidy increase, thought to be crucial for progression through meiosis and formation of diploid gametes in parthenogenetic lizards, is not observed in the majority of primary oocytes regardless of the animal's age.

### Characterization of prophase I in *Aspidoscelis*

**Fig. 2.**
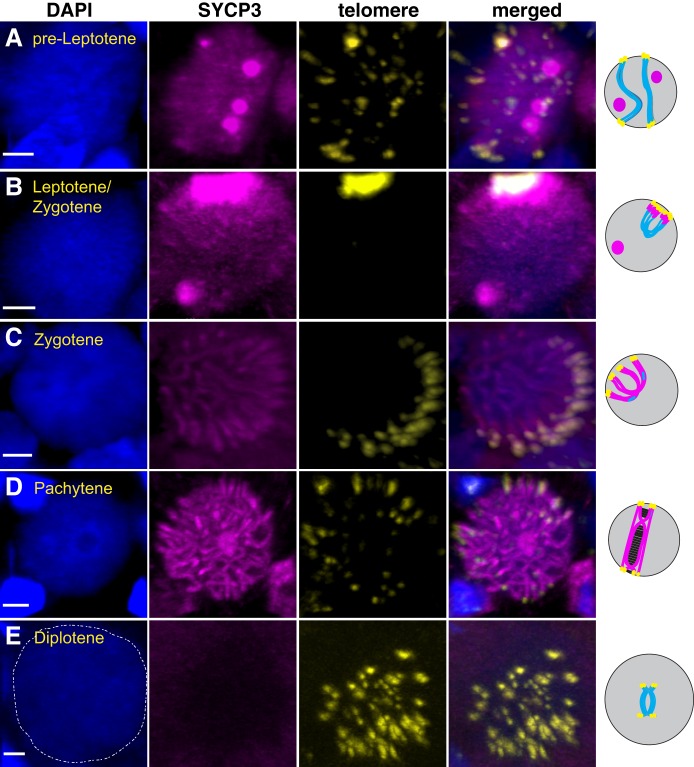
**Stages of meiotic prophase in *A. inornata.*** Germinal bed stained with DAPI (blue), anti-SYCP3 (magenta) and telomere FISH (yellow). Stages were assigned based on SYCP3 staining pattern, telomere position and cell size. Representative examples for (A) pre-leptotene, (B) late leptotene/early zygotene, (C) zygotene, (D) pachytene, and (E) early diplotene. The diplotene nucleus is outlined with a dashed white line in the DAPI image. Scale bars: 2 µm. Schematics of prophase nuclei at the respective stages are shown to the right of the microscopic images showing DNA in blue, telomeres in yellow and SYCP3 in magenta.

To gain further insights into progression through prophase we needed to examine the individual stages of meiotic prophase in bisexual and parthenogenetic animals. In bisexual *A. inornata*, immunostaining for SYCP3 combined with fluorescent *in situ* hybridization (FISH) for telomeres revealed a category of cells with nuclei ranging in size from 8 to 10 µm displaying one or more SYCP3 foci and telomeres that ranged from nucleoplasm-dispersed to nuclear periphery-localized (Fig. 2A), as observed during pre-leptotene for bovine oocytes (Pfeifer et al., 2003). Nuclei with the most tightly clustered telomeres showed an overlapping aggregation of SYCP3, but lacked threads of SYCP3-positive axial elements extending from the telomere bouquet, indicating that telomere clustering precedes SYCP3 localization along the length of chromosomes in this species (Fig. 2B). We refer to oocytes with tight telomere bouquets as leptotene/zygotene. Zygotene-stage oocytes were assigned based on SYCP3-positive threads extending from peripherally localized telomeres into the interior of the nucleus (Fig. 2C). At this stage, SYCP3 staining was most intense near the telomeres, consistent with pairing initiating near chromosome ends as recently also reported for planaria (Xiang et al., 2014). Cells in which telomeres were dispersed and SYCP3 antibodies stained well-defined and extended threads were categorized as pachytene (Fig. 2D). Defined SYCP3 staining and a characteristic pattern of telomere localization are absent in early diplotene, but cells in this stage can easily be assigned based on their size of 20 to 40 µm clearly exceeding the size of all other cells in the germinal bed (Fig. 2E; Fig. S2).

Combining telomere FISH with SYCP3 staining for *A. neomexicana* (parthenogenetic) identified nuclei in each of the stages described above (Fig. S3), although the diplotene stage was exceedingly rare (see below). A subset of 13 *A. neomexicana* chromosomes harbor large tracts of internal telomeric sequences (ITS) that stain considerably more brightly by telomere FISH than the telomeric repeats at chromosome ends (Fig. S4A) (Lutes et al., 2010). Interestingly, we found that the ITSs are consistently excluded from the telomere bouquet (Fig. S4B).

### Accumulation of oocytes at the pairing stage in parthenogenetic animals

**Fig. 3.**
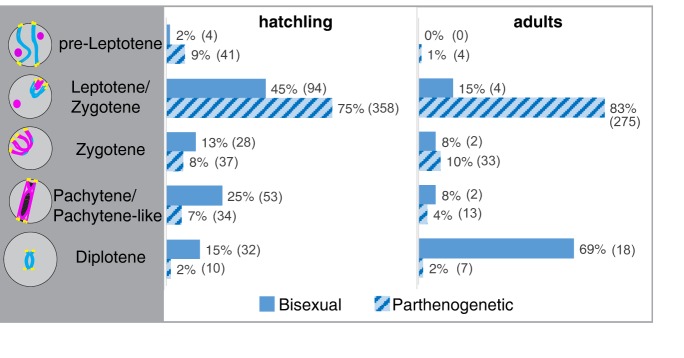
**Distribution of oocytes across prophase I in hatchlings and adults.** Meiotic stages were assigned based on anti-SYCP3 staining and telomere localization in newly hatched and adult *A. inornata* (solid blue) and *A. neomexicana* (striped). Number of oocytes assigned to specific stages: 211 (hatchling *A. inornata*), 480 (hatchling *A. neomexicana*), 26 (adult *A. inornata*), 332 (adult *A. neomexicana*). Absolute numbers of oocytes in each class are shown in parentheses.

We next used the SYCP3-telomere assay on germinal beds from newly hatched animals to assign oocytes to specific stages (Fig. 3). Out of 5131 cells examined in GBs isolated from four bisexual *A. inornata*, 223 (4.4%) were oocytes (Fig. S5). Of these, 211 were unambiguously assigned to specific stages of meiotic prophase with zygotene being most prevalent (45%; Fig. 3). Evaluation of 11,513 germinal bed cells from nine hatchling *A. neomexicana* (parthenogenetic) yielded 521 oocytes (4.5% of total), of which 480 were unambiguously staged. As in the bisexual species, zygotene was the most abundant stage (75%).

We next assembled meiotic stage profiles for adult lizards. Contrary to the similar profiles for newly hatched animals, a clear difference was observed between bisexual and parthenogenetic animals. Of 4199 germinal bed cells from three bisexual adults, only 27 oocytes (0.64% of total) were identified. Of 26 unambiguously staged oocytes 18 (69%) were in diplotene (Fig. 3). The one oocyte not confidently staged most closely resembled diplotene as well. The number of oocytes in the GB is therefore ∼eightfold reduced in adults compared with hatchlings, and most of the surviving oocytes have progressed into diplotene. In contrast, germinal beds of five adult *A. neomexicana* yielded a total of 8902 cells, of which 398 were identified as oocytes (4.5% of total) and 332 were assigned to specific stages (Fig. 3). Strikingly, 83% of these were in zygotene and only 2% were in diplotene. The 66 oocytes that could not be unambiguously staged most closely resembled zygotene and pachytene, and none were in diplotene. Nevertheless, the ovaries of all adult animals used in this analysis contained between 3 and 18 previtellogenic follicles next to the GBs, confirming that all animals had reached sexual maturity. The presence of large numbers of early-stage oocytes seen in parthenogenetic, but not the bisexual, adults is therefore a notable difference.

### Structure illumination microscopy reveals pairing defect

**Fig. 4.**
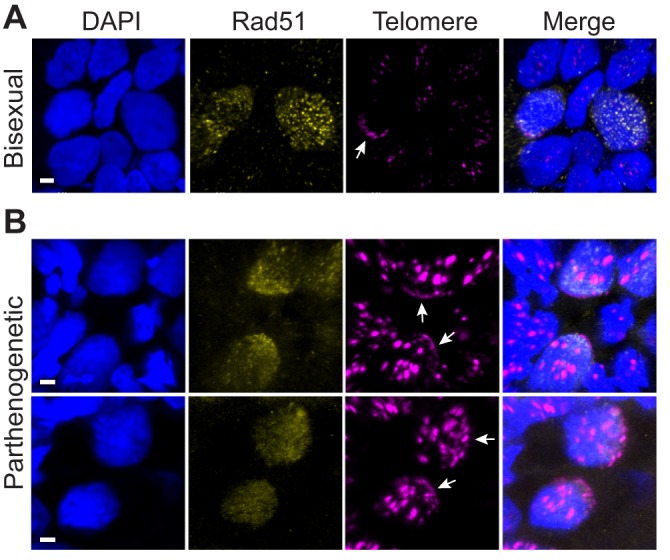
**Rad51 localization in oocytes.** Germinal beds from bisexual *A. inornata* (A) and parthenogenetic *A. neomexican*a (B) were stained with DAPI (blue), anti-Rad51 (yellow) and telomere FISH (magenta). Telomere bouquets are indicated with white arrows. The chromosome internal arrays of telomeric sequence found on 13 chromosomes in *A. neomexicana* do not contribute to the meiotic bouquet. Scale bars: 2 µm.

We speculated that structural differences and sequence divergence between ancestrally homologous (homeologous) chromosomes in the parthenogenetic species might cause arrest at the pairing stage for those oocytes that have not undergone the additional doubling in DNA content. To examine whether recombination takes place in 4C oocytes, we assayed for Rad51 foci in combination with telomeric FISH to assist in staging of oocytes. In GBs from bisexual animals, Rad51 foci were readily detected in cells with clustered telomeres (Fig. 4A). In contrast, Rad51 staining was diffuse in oocytes from parthenogenetic females (Fig. 4B), an observation consistent with failure to initiate homologous pairing and strand exchange. Whereas the absence of Rad51 foci is an indication of failure to recombine, it is not a direct measure of the extent of homolog pairing in 4C oocytes.

**Fig. 5.**
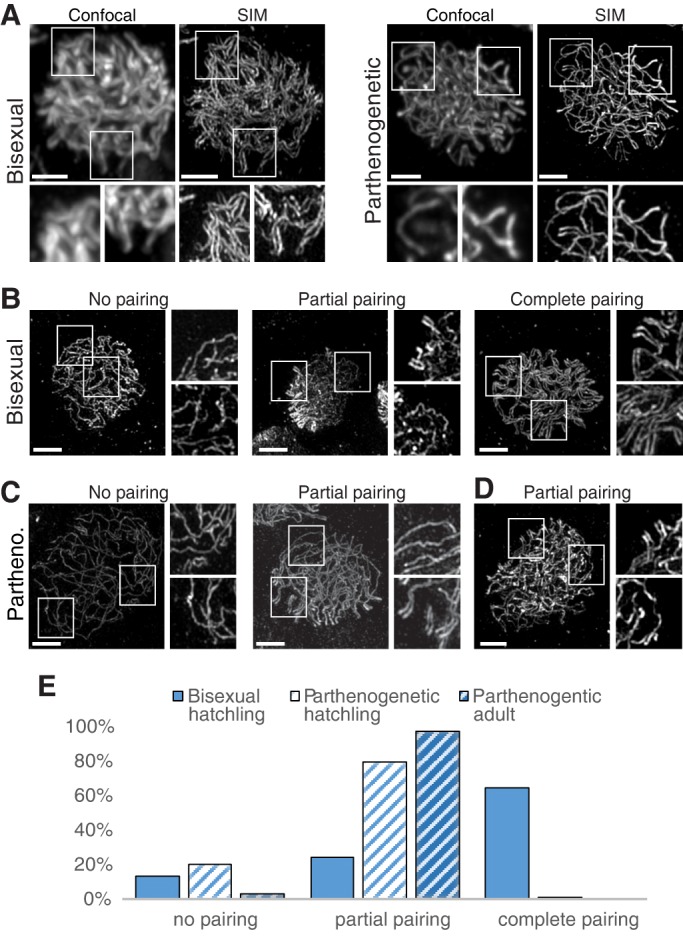
**Structured illumination microscopy (SIM) of SYCP3 threads resolves lateral elements on paired chromosomes.** (A) Germinal beds were stained with anti-SYCP3 and the same oocytes were imaged by confocal microscopy (left) and SIM (right). The boxed areas in each image are shown enlarged below the main images. (B) Examples of oocytes with unpaired, partially paired and fully paired chromosomes from *A. inornata* hatchling. (C) Unpaired and partially paired chromosomes from *A. neomexicana* hatchling. (D) Example of partially paired chromosomes from an adult *A. neomexicana*. (E) Quantification of pairing in hatchling *A. inornata* (solid blue bar; *n*=85), hatchling (light blue stripes; *n*=115) and adult (dark blue stripes; *n*=33) *A. neomexicana*. Scale bars: 2 µm.

As a structural component of the axial elements of partially condensed chromosomes, thread-like SYCP3 staining is observed prior to pairing (Dobson et al., 1994; Pfeifer et al., 2003). As chromosomes synapse, the axial elements become the lateral elements of the SC. At this stage the lateral elements are less than 200 nm apart and cannot be resolved into individual threads by conventional light microscopy. The parallel threads of SYCP3 that signify synapsed chromosomes can therefore not be distinguished from the single threads that precede pairing. Structured illumination microscopy (SIM) overcomes this limitation and has been demonstrated to resolve parallel tracks of SYCP3 staining in other species (Gustafsson et al., 2008). Indeed, images of oocytes that appear similar by confocal microscopy show distinct single and double tracks by SIM imaging, representing unpaired and paired chromosomes (Fig. 5A). Examination of parallel tracks near chromosome ends where they were present in both species, revealed a center distance of 196 nm and 193 nm for *A. inornata* and *A. neomexicana*, respectively (Fig. S6), similar to super-resolution measurements in murine oocytes (Prakash et al., 2015).

We took advantage of the ability to distinguish between unpaired and synapsed chromosomes by SIM and assessed the abundance of single versus parallel tracks (Fig. 5B,C). Partial pairing was generally observed near chromosome ends (see insets for center panels in Fig. 5B,C), consistent with the accumulation of SYCP3 at telomeres (Fig. 2B,C) and the notion that pairing initiates at chromosome ends and progresses internally from there. For bisexual *A. inornata* complete pairing was observed for 54 of 85 oocytes; 20 showed partial pairing and 11 contained only single tracks of SYCP3 staining (Fig. 5B,E). In contrast, 91 of 115 oocytes from parthenogenetic *A. neomexicana* showed pairing restricted to chromosome ends, whereas 23 showed no pairing at all and only a single oocyte showed extensive parallel tracks. To verify that the low incidence of paired chromosomes in hatchling *A. neomexicana* was not simply an indication of delayed progression through prophase, we examined 33 oocytes from a parthenogenetic adult. These showed overwhelmingly partial pairing and one oocyte showed no pairing at all (Fig. 5D,E). In summary, these results strongly suggest that the vast majority of oocytes in *A. neomexicana* enter meiosis with a 4C DNA content and pairing of homeologous chromosomes is impaired. This results in the accumulation of zygotene-arrested oocytes in parthenogenetic adults. Some oocytes in *A. neomexicana* had a zygotene- or pachytene-like appearance based on telomere localization and SYCP3 staining, but nevertheless showed only telomere-proximal chromosome pairing.

### Effect of meiotic defect on fecundity

**Fig. 6.**
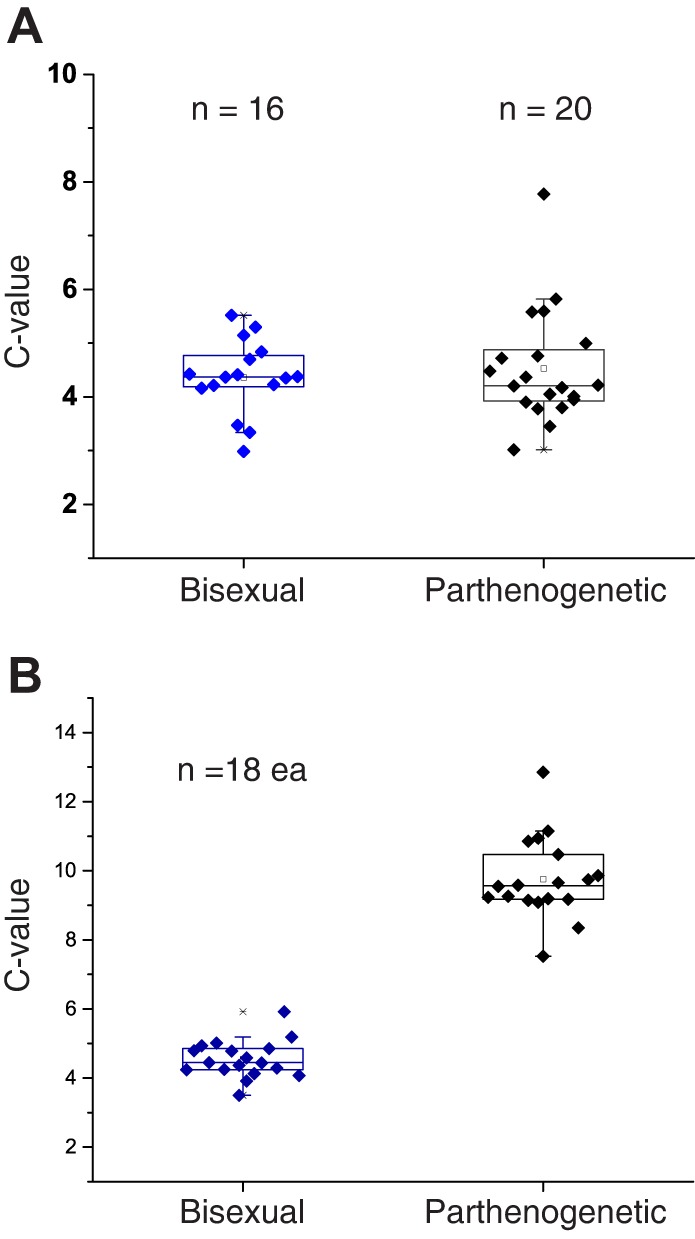
**DNA content analysis for pachytene-like and diplotene oocytes.** (A) DNA content analysis of pachytene-like oocytes based on SYCP3 and telomere localization. DNA content was measured based on nuclear DAPI fluorescence for each oocyte relative to at least three surrounding stromal cells assumed to be in G1/G0. DNA content differences are not significant [*P*=0.585 one-way analysis of variance (ANOVA) test]. Germinal beds were isolated from hatchling of *A. inornata* (bisexual) and *A. neomexicana* (parthenogenetic). (B) Image cytometry as in A for early diplotene oocytes identified based on cell morphology and size, as well as absence of anti-SYCP3 staining and telomere clustering. DNA content differences between the two species are highly significant [*P*<2×10^−16^ one-way analysis of variance (ANOVA) test].

In an attempt to resolve the paradox of almost all zygotene- and pachytene-like oocytes in *A. neomexicana* harboring half as much DNA as previously observed in diplotene, we quantified the DNA content specifically for those oocytes that seemed to be in pachytene and early diplotene. With rare exceptions, pachytene-like oocytes from parthenogenetic *A. neomexicana* had a 4C DNA content indistinguishable from bisexual *A. inornata* (Fig. 6A). In contrast, oocytes classified as diplotene contained twice as much DNA in parthenogenetic compared with bisexual animals (Fig. 6B). These results obtained for early diplotene cells residing in the germinal bed match the DNA quantification for previtellogenic follicles in mid and late diplotene where pairing of identical chromosomes has been demonstrated (Lutes et al., 2010).

**Fig. 7.**
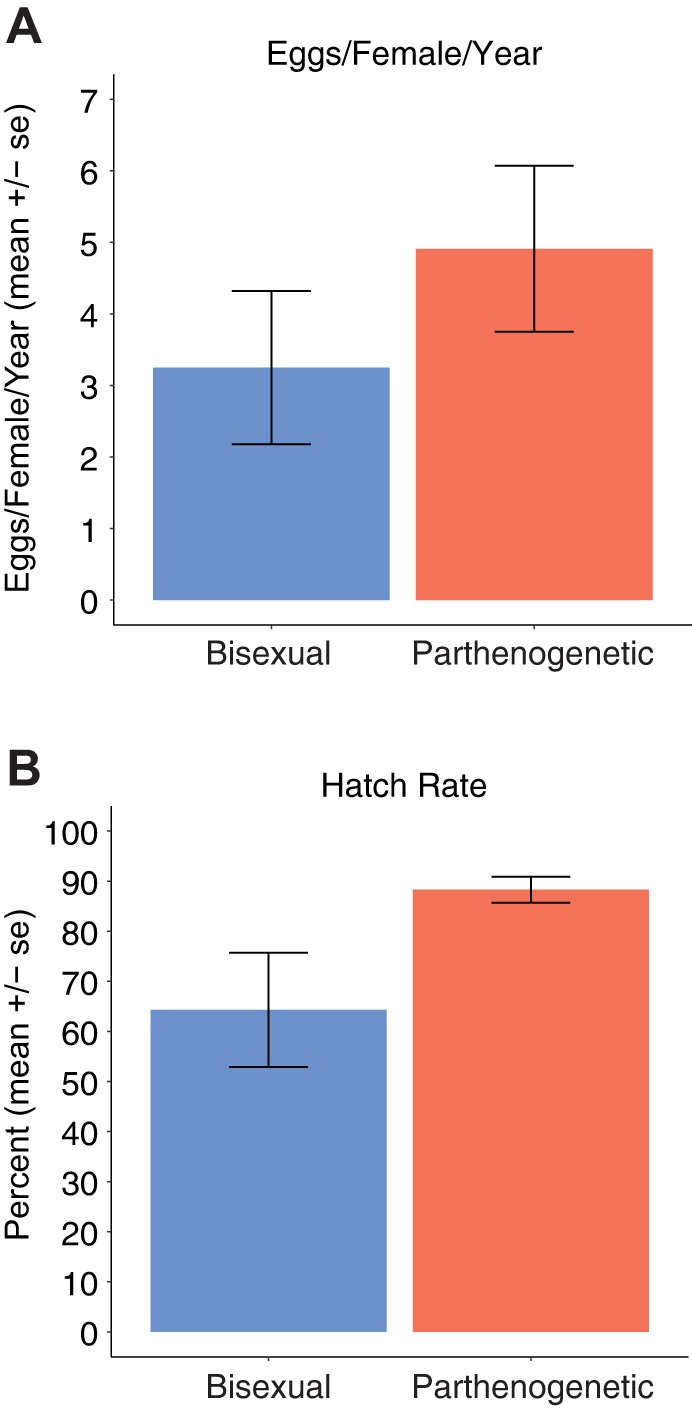
**Fecundity and hatch rates for *A. inornata* (bisexual) and *A. neomexicana* (parthenogenetic).** (A) Fecundity based on the number of eggs recovered from pens collectively housing 37 female and 24 male *A. inornata* and 57 *A. neomexicana*, respectively*.* As not all animals were present for the full 365 day collection period, the number of eggs recovered was divided by the sum of days for all females in the experiment (10169, *A. inornata*; 17490, *A. neomexicana*) and normalized to one year. Mean and standard errors were calculated by treating animals housed in separate pens as biological replicates. (B) Hatch rate for eggs collected in A after incubation at 28°C for ∼2 months. Some late-stage embryos (28 for *A. neomexicana* and 1 for *A. inornata*) were used in research and those eggs were excluded from the analysis.

The abundance of oocytes that enter meiosis without prior endoreplication was surprising and indicated that the meiotic mechanism that permits parthenogenetic reproduction relies on a rare and perhaps stochastic event rather than two coordinated rounds of replication preceding entry into prophase I. As a consequence, one might expect that the fecundity of parthenogenetic females is significantly reduced compared with bisexual counterparts. However, monitoring of 57 *A. neomexicana* and 37 *A. inornata* females in our laboratory colony revealed that parthenogenetic animals lay no fewer eggs over the course of a year than bisexual females (Fig. 7A). Furthermore, the hatch rate for *A. neomexicana* was higher than for *A. inornata*, possibly reflecting incomplete fertilization of *A. inornata* clutches despite cohousing of all females with conspecific males (Fig. 7B). In any case, there is no indication that fecundity of parthenogenetic animals is impaired despite the majority of oocytes entering a non-productive meiosis in the absence of endoreplication.

## DISCUSSION

The observations that previtellogenic follicles isolated from parthenogenetic lizards contain twice as much DNA as those from closely related bisexual species supported a model in which meiosis is preceded by endoreplication. However, we have now shown that oocytes in parthenogenetic *A. neomexicana* overwhelmingly enter meiosis in the diploid state. Among hundreds of oocytes examined in early prophase not one was found to harbor an 8C DNA content. Oocytes with a 4C DNA content enter meiotic prophase but fail to stably pair homeologous chromosomes and arrest at the pairing stage. Asynapsis and arrest are commonly observed in first generation interspecific hybrids even between closely related subspecies such as *Mus musculus musculus* and *M. m. domesticus* (Bhattacharyya et al., 2013).

*Aspidoscelis* *neomexicana* and its two bisexual progenitor species *A. marmorata* (formerly *A. tigri*s *marmorata*) and *A. inornata* each have 46 chromosomes, but the size distribution and overall architecture are quite distinct (Cole et al., 1988; Lowe and Wright, 1966). *A. marmorata* harbors three pairs of large metacentrics, eight pairs of medium sized submeta- to subtelocentric and 12 pairs of microchromosomes. In contrast, only one pair of large metacentric chromosomes is present in *A. inornata* together with 12 pairs of subtelocentric and telocentric medium-sized chromosomes and 10 pairs of microchromosomes. Although two simple fission events might explain the conversion of two large metacentrics into four smaller telocentric chromosomes, the difference in the number of microchromosomes indicates that more complex rearrangements have led to the distinct karyotypes between the two *Aspidoscelis* species that hybridized to produce *A. neomexicana* (Reeder et al., 2002). Further evidence for differences in chromosomal architecture comes from *in situ* hybridization with repeat probes that revealed large arrays of CCAAGG and GGGTTA repeats on multiple chromosomes in *A. neomexicana* (Lutes et al., 2010). Hybridization of metaphase spreads from *A. marmorata* and *A. inornata* demonstrated that these repeat arrays were readily detected in *A. marmorata*, but not *A. inornata*, thereby accounting for additional structural variation between the *A. marmorata*- and *A. inornata*-derived chromosomes in the hybrid species. In light of these gross chromosomal differences between the two parental sets of chromosomes, it is not surprising that homeolog pairing and progression through meiosis fail when homeologous chromosomes are the only available pairing partner.

Despite the vast majority of oocytes entering meiosis with a 4C DNA content and accumulating at the pairing stage, a small number of cells evidently progress to form mature diploid eggs. It is presently unclear how these 8C oocytes arise. Premeiotic endoreplication or oogonial fusion might occur at low frequency and rapid progression through the early stages of prophase I might have precluded their detection until the later stages. Alternatively, the accumulation of large numbers of 4C oocytes might increase the opportunities for rare fusion events generating 8C oocytes during prophase. Such fusions have been proposed to contribute to the origin of triploidy in humans (Hardarson et al., 2002; Zaragoza et al., 2000). It is an intriguing possibility that events such as oocyte fusion occur as infrequent errors during normal oogenesis in many species, but come under positive selection in hybrids where reproduction depends on pseudo-tetraploid oocytes.

In the laboratory colony that contributed to this study, the fecundity of parthenogenetic *A. neomexicana* compared favorably with that of *A. inornata*, even though the overwhelming majority of *A. neomexicana* oocytes lacked the extra set of chromosomes necessary for completing meiosis. Evidently, the event that generates pseudo-tetraploidy occurs with sufficient frequency to sustain natural populations of *A. neomexicana* over many generations; its abundance in New Mexico and along the Rio Grande in Texas justifies its IUCN listing as a Species of Least Concern (The IUCN Red List of Threatened Species, v.2015-4, downloaded on 23 May 2016; http://www.iucnredlist.org). The reservoir of oocytes in *A. neomexicana* is deep enough to tolerate an extremely low rate of progression through meiosis with little or no impact on the number of mature eggs that are produced and deposited.

## MATERIALS AND METHODS

### Specimens

All animals used in this study were produced in the AAALAC-accredited Stowers Reptile and Aquatics Facility in compliance with protocols approved by the Institutional Animal Care and Use Committee. Breeding colonies were established from specimens collected in New Mexico under permit numbers 3199 and 3395 (*A. inornata*, *A. marmorata* and *A. neomexicana*) and Arizona under license number SP564133 (*A. arizonae*). For the purpose of this study we make no distinction between *A. inornata* and *A. arizonae*, the latter being considered a subspecies of *A. inornata* by some and not distinguishable from geographically proximate *A. inornata* based on diagnostic morphological features (Sullivan et al., 2013).

### SYCP3 antibody

A sequence alignment of SYCP3 mRNA from *Gallus gallus*, *Anolis carolinensis*, *Xenopus tropicalis* and *X. laevis* was used to design degenerate primers 5′-RGCKGAYATYARYAARGCTC-3′ and 5′-CYTGCTKTTGDGTGTCCATC-3′ (Integrated DNA Technologies). *Aspidoscelis gularis* testes total RNA was reverse transcribed using the Superscript First-Strand Synthesis for RT-PCR kit (Invitrogen) followed by PCR in 25 µl reactions (1 µl of 1:40 dilution cDNA; 1× Pfu buffer; 0.4 mM dNTP mix; 1 µM each primer; 5% DMSO and 1.25 U Pfu Hotstart Turbo polymerase, Agilent Technologies) under the following conditions: 3 min at 94°C; 35 cycles of 30 s at 94°C, 1 min at 44°C, 1 min at 72°C; 5 min at 72°C. Products were purified by gel electrophoresis and sequence verified. Subsequently, the 5′ and 3′ ends of SYCP3 cDNA were cloned using the Marathon cDNA Amplification kit (Clontech) and gene-specific primer pairs in a nested PCR. For the 5′ end, the following primers were used: 5′-GAGCCTTCTCCATGTCCTCCATACTC-3′ (outer primer) and 5′-TGCTGTTGTCGAAACATGTTCGCCAG-3′ (inner primer). To clone the 3′ end: 5′-ACTGGCGAACATGTTTCGACAACAGC-3′ (outer primer) and 5′-TATGGAGGACATGGAGAAGGCTCATG-3′ (inner primer). The PCR conditions were as follows for the first round of nested PCR: 1 µl of 1:100 dilution cDNA, 1× Thermopol buffer (New England BioLabs), 0.4 mM dNTP mix, 0.4 µM each of respective outer primer and Adaptor Primer 1, and 2.5 U Taq polymerase. The PCR program: 1 min 94°C; 35 cycles of 30 s at 94°C, 2 min at 68°C. A 1 µl aliquot of PCR product was used for the second round of nested PCR with the inner primer and Adaptor Primer 2. After gel electrophoresis, bands of expected size were cut out, gel purified (Qiagen) and sequenced. To clone full-length SYCP3, the primers 5′-TGGGCGCCTAAAAGGAGGAG-3′ and 5′-AAACACTCTTTTGACCCTTCATGG-3′ were used in the following PCR: 1 µl of 1:10 cDNA from Marathon kit described above, 1× Pfu buffer, 0.4 mM dNTP mix, 1 µM each primer, and 1.25 U Pfu Hotstart Turbo polymerase. The PCR program: 3 min at 94°C; 30 cycles of 30 s at 62°C, 1.5 min at 72°C; 5 min at 72°C.

For purification, the full-length *A. gularis* SYCP3 ORF was amplified to include *Sac*I and *Eco*RI restriction sites and subsequently cloned into a pVCH6 expression vector and transformed into BL21 Rosetta (DE3) pLysS *Escherichia coli* cells (EMD Millipore). The His-tagged protein was purified under denaturing conditions using a HiTrap Chelating HP column (GE Healthcare) on an ÄKTA FPLC system. The protein was eluted from the column with a 4-100% gradient of 50 mM Tris-Cl pH 7.5, 1 M NaCl, 500 mM imidazole, 6 M urea, 0.1% Triton X-100. Eluates containing a single band based on Coomassie-stained SDS-PAGE were pooled and dialyzed in 50 mM sodium phosphate buffer pH 7 for 4 h in a Slide-A-Lyzer dialysis cassette, 10 K MWCO (Thermo Scientific) at 4°C. Samples were then concentrated using a Centriplus or Centricon tube (Millipore). Polyclonal antibodies were raised in rabbits (Yenzym Antibodies, LLC, South San Francisco, CA).

### Immunofluorescence (IF) and fluorescence *in situ* hybridization (FISH)

Germinal beds were isolated in phosphate buffered saline (PBS), pH 7.4 and transferred to 4% paraformaldehyde (PFA) in PBS for 45-60 min, then washed twice for 5 min in PBS (400 µl) and blocked in 1% bovine serum albumin (BSA) + 0.2% Triton X-100 in PBS for 2 h. Samples were then incubated 4 to 20 h in primary antibody in PBS (1% BSA), anti-SYCP3 (1:500; clone YZ3150 lab stock); anti-hRad51 (1:500; gift from S. West, Francis Crick Institute, London, UK). Samples were then washed four times for 30 min with 400 µl PBS (1% BSA) and incubated overnight in secondary antibody (1:300 Alexa Fluor 546 goat anti-rabbit, Life Technologies, cat. no. A11035 or 1:300 Alexa Fluor 488 goat anti-rabbit, Life Technologies, cat. no. A11008) at 4**°**C. After four 30 min washes in 1% BSA/PBS, germinal beds were incubated overnight in 1 µg/ml 4′,6-diamidino-2-phenylindole (DAPI). The following day, these were mounted in PBS containing 1 µg/ml DAPI in a poly-d-lysine-coated glass bottom dish (MatTek, P35GC-1.5-14-C) by applying a small ring of vacuum grease to the bottom of the dish and adding PBS/DAPI solution to the inside of the ring. Germinal beds were immersed in the solution, and a 22×22 mm coverslip was gently placed on top of the ring to reduce evaporation during imaging.

For combined IF and FISH, germinal beds were first stained for either SYCP3 or hRad51 as described above except that prior to DAPI incubation the samples were washed briefly in buffer A (15 mM PIPES pH 6.8, 20 mM NaCl, 60 mM KCl, 0.5 mM EGTA, 2 mM EDTA, 0.5 mM spermidine, 1 mM DTT) and then treated with RNase A (50 µg/ml) in buffer A for 1 h at 37°C, then washed with buffer A and fixed in 4% PFA/Buffer A for 1 h at room temperature. Afterwards, the germinal beds were incubated in 2× SSCT (0.3 M NaCl, 34 mM trisodium citrate, 0.1% Tween-20, pH 7.0) for 30 min then taken through a 20%, 40%, 50% formamide + 2× SSCT series with 20 min incubations in each solution. The samples were then placed in PCR tubes containing 40 µl hybridization mix: 70% formamide, 10% dextran sulfate, 3× SSC and 20 nM of a 5′-labeled Alexa Fluor 546 locked nucleic acid (LNA) probe 5′-(CCCTAA)_3_-3′ (Exiqon). Samples were incubated in an Eppendorf thermocycler with the following program: 22°C for 6 h, 40°C for 30 min, 85°C for 2 min and 12-20 h at 37°C. Samples were washed three times in 50% formamide + 2× SSCT followed by washes in 25% formamide + 2× SSCT, 2× SSCT, and overnight incubation in DAPI (1 µg/ml) in PBS. The following day, the samples were mounted and imaged.

### Microscopy

Samples were imaged using a LSM 510 META (Carl Zeiss Jena) system equipped with a C-Apochromat 40×/1.20 W Corr M27 (WD=0.17 mm) or a Plan-Apochromat 63×/1.40 Oil DIC M27 (WD=0.19 mm) objective (Carl Zeiss Jena). The Ti:Sapphire 720-930 (Coherent Chameleon Ultra), 458, 488, 514 Ar-Ion 30 mW (Lasos LGK 7812 ML4, 577009-2125-000) and 561 DPSS 15 mW (Melles Griot, 85-YCA-010) lasers were used to excite the fluorescent dyes. DAPI was visualized by two-photon excitation at 750 nm to avoid out-of-focus bleaching, and fluorescence emission was collected between 390 and 465 nm. For IF and FISH, excitation at 488 or 561 was used depending on the conjugated dye and emission was collected between 505 and 550 nm, LP 575 nm and 710 nm, respectively. Images were processed in Imaris (Bitplane) or Image J (NIH). Maximum projections of a subset of the raw data stacks are shown. A Gaussian blur with a radius of 0.8 pixels (ImageJ) or a 3×3×3 Median filter (Imaris) were applied in some cases.

### DNA quantification

Image stacks from DAPI-only or DAPI, anti-SYCP3 and telomere FISH germinal beds were analyzed using Imaris 7.7.0 software. The Surface function was used to automatically identify and encompass nuclei in the DAPI channel. Subsequently, the DAPI intensity sum was calculated for each object. Nuclei that based on visual inspection had not been accurately outlined with the Surface function were excluded from the analysis. In DAPI-only samples, nuclei were categorized as ‘non-oocytes’ and ‘oocytes’ based on nuclear and cellular morphology. For each image stack, the intensity sum values of non-oocytes were plotted. The majority of nuclei displayed similar intensity sum values and were assumed to be diploid G1 and G0 cells. The average of these values was set as 2C. The C-values for all nuclei, non-oocyte and oocyte, were then determined relative to this 2C standard. Oocytes processed with anti-SYCP3 and telomere FISH were further subcategorized into the stages of leptotene, zygotene, zygotene-pachytene, pachytene and diplotene.

To analyze meiotic progression, C-values for individual pachytene and diplotene oocytes were calculated using AIM (Zeiss) and ImageJ software. The oocytes, as well as three neighboring somatic cells, were individually outlined using AIM software and the DAPI intensity of three neighboring nuclei was measured in ImageJ. The average intensity sum of the neighboring nuclei was set as the 2C standard and from it, the C-value of the oocyte was calculated.

### SIM imaging

Lizard germinal beds were fixed in 100% methanol at 4°C for 1 h and immunostained with anti-SYCP3 as described above. The GBs were rinsed well with 1×PBS, dehydrated through 30% sucrose and followed by embedding with OCT compound (Tissue-Tek). Cryo sections with 15 µm thickness were cut using a Leica CM3050S cryostat (Leica Biosystems) and mounted on 22×22 glass coverslips for SIM imaging. SIM images were acquired with an Applied Precision OMX Blaze (GE Healthcare). A 60×1.42 numerical aperture Plan Apo oil objective was used, and emission photons were collected onto three PCO Edge sCMOS cameras (PCO AG), each dedicated to one specific channel. Color alignment of *x*-*y* direction was performed using the color alignment slide provided by GE Healthcare, and axial direction was calibrated by 100 nm TetraSpeck beads (Life Technologies). SIM images were reconstructed with the Applied Precision software Softworx with a Wiener filter of 0.001. For image preparation, reconstructed images were scaled 2×2 with bilinear interpolation then smoothed with a Gaussian blur with a 0.8 pixel radius.
